# Enzymatic Glyco-Modification of Synthetic Membrane Systems

**DOI:** 10.3390/biom13020335

**Published:** 2023-02-09

**Authors:** Dylan Jabeguero, Lina Siukstaite, Chunyue Wang, Anna Mitrovic, Serge Pérez, Olga Makshakova, Ralf P. Richter, Winfried Römer, Christelle Breton

**Affiliations:** 1 Centre de Recherches sur les Macromolécules Végétales (CERMAV), CNRS, University Grenoble Alpes, 38041 Grenoble, France; 2 Faculty of Biology, Signalling Research Centres BIOSS and CIBSS, Freiburg Institute of Advanced Studies (FRIAS), Albert-Ludwigs-University Freiburg, 79104 Freiburg, Germany; 3 School of Biomedical Sciences, Faculty of Biological Sciences, School of Physics and Astronomy, Faculty of Engineering and Physical Sciences, Astbury Centre for Structural Molecular Biology, Bragg Centre for Materials Research, University of Leeds, Leeds LS2 9JT, UK; 4 FRC Kazan Scientific Center of RAS, Kazan Institute of Biochemistry and Biophysics, 420111 Kazan, Russia

**Keywords:** giant unilamellar vesicles, globotriaosylceramide, LgtC, molecular dynamics, supported lipid bilayer

## Abstract

The present report assesses the capability of a soluble glycosyltransferase to modify glycolipids organized in two synthetic membrane systems that are attractive models to mimic cell membranes: giant unilamellar vesicles (GUVs) and supported lipid bilayers (SLBs). The objective was to synthesize the Gb3 antigen (Galα1,4Galβ1,4Glcβ-Cer), a cancer biomarker, at the surface of these membrane models. A soluble form of LgtC that adds a galactose residue from UDP-Gal to lactose-containing acceptors was selected. Although less efficient than with lactose, the ability of LgtC to utilize lactosyl–ceramide as an acceptor was demonstrated on GUVs and SLBs. The reaction was monitored using the B-subunit of Shiga toxin as Gb3-binding lectin. Quartz crystal microbalance with dissipation analysis showed that transient binding of LgtC at the membrane surface was sufficient for a productive conversion of LacCer to Gb3. Molecular dynamics simulations provided structural elements to help rationalize experimental data.

## 1. Introduction

The dense layer of glycoconjugates (glycolipids, glycoproteins and proteoglycans) attached on the surface of various cell types, called the glycocalyx, is an information-rich barrier that mediates many molecular interactions in cell–cell communication, recognition, adhesion, signal transduction and host–pathogen interactions [1,2,3,4] Aberrant cell surface glycosylation is a feature of many cancers, and many tumor-associated carbohydrate antigens are attractive targets for applications in diagnostics, drug delivery and targeted immunotherapy [5,6,7,8]

Synthetic glycobiology is an emerging field that contributes significantly to our understanding of cell surface glycans’ biological roles and impacts diagnostic and therapeutic applications through the development of innovative methods to manipulate cell surface glycosylation [9,10]. Glycoconjugates are synthesized by the concerted action of ~200 glycosyltransferases (GTs) that reside in the endoplasmic reticulum and the Golgi apparatus [11,12]. Glycocalyces can therefore be modified genetically. However, perturbing the biosynthetic machinery can have undesirable effects, such as cellular lethality or no observable effect due to functional redundancy. Chemoenzymatic methods and de novo glycocalyx remodeling have therefore attracted much attention. They allow a precise modification of glycans and are based either on incubating cells with exogenously added carbohydrate-active enzymes or on the passive insertion of functionalized glycolipids into the plasma membrane [13,14,15].

Novel strategies have been developed to construct artificial glycocalyces [16]. Synthetic glycoconjugates, whose architecture and functionality can be molecularly defined and tuned, are promising tools for studying some aspects of the complexity and organization of the native glycocalyx [17]. Their integration into model membrane systems is an interesting approach to recreating complex cellular processes (e.g., endocytosis) or forming proto-tissues. Giant unilamellar vesicles (GUVs) and supported lipid bilayers (SLBs) offer attractive models to mimic cell membranes [18,19]. They have provided insight into the underlying mechanisms of cell adhesion and the signaling processes that occur at or across the lipid membrane [20,21,22]. Glycocalyx reconstruction requires the decoration of synthetic membranes with selected and controlled glycoconjugates. Although within the toolbox of synthetic glycobiology, GTs have been used mainly in a cellular context [9,10,13,14], but curiously very little on synthetic membrane systems. One example is illustrated by the use of the bovine β4-galactosyltransferase to glycosylate an N-acetylglucosamine-capped glycolipid [23]. The authors observed that the enzyme reaction rate was significantly increased upon lipid clustering, highlighting the sensitivity of GT activity to details of the membrane microenvironment. Another example utilized a hybrid bilayer, consisting of a self-assembled alkanethiol monolayer (SAM) and a lipid monolayer, in conjunction with quartz crystal microbalance with dissipation (QCM-D) for real-time monitoring of lipid mannosylation by α3-mannosyltransferase (GumH) [24].

**Figure 1:**
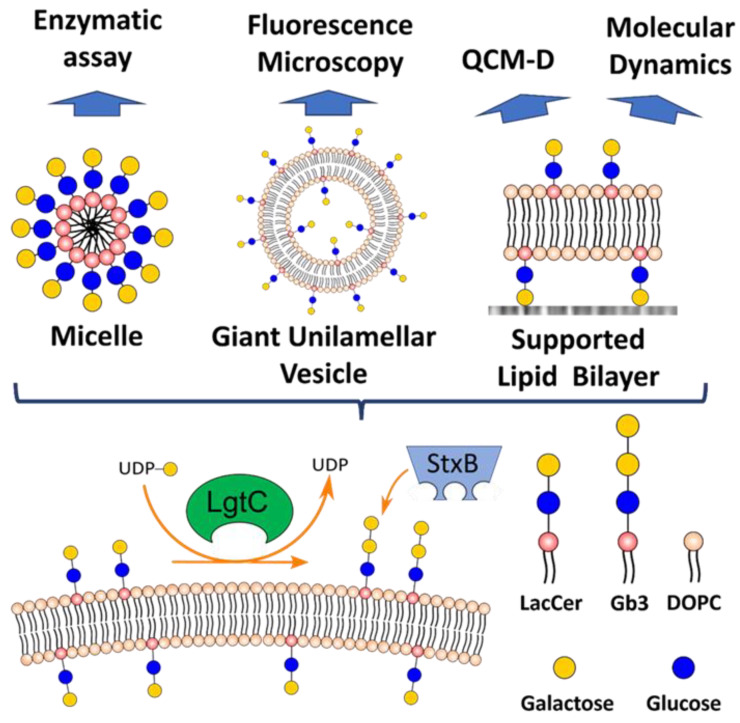
Overview of the LgtC enzyme reaction, different lipid constructs and the techniques utilized.

The present report assesses the capability of soluble GTs to glycosylate (glyco)lipids in several membrane contexts—micelles, GUVs and SLBs (Figure 1). Although most GTs are membrane-bound in vivo, it is often possible to express their catalytic domains in a soluble form [25], thus potentially facilitating their practical application in engineering artificial glycocalyces.

The soluble GT selected for this study was the bacterial galactosyltransferase (LgtC) from *Neisseria meningitidis*, which is involved in the synthesis of lipooligosaccharides [26]. LgtC catalyzes the transfer of galactose from UDP-Gal to lactose-containing acceptors, forming an α1,4-linkage. We investigated the ability of LgtC to add a galactose residue to lactosyl–ceramide (LacCer) to form the globotriaosylceramide (Gb3) glycosphingolipid (Galα1,4Galβ1,4Glcβ-Cer). Gb3 is known as a host cell receptor used by several pathogens. It is also overexpressed in several cancers, making it a target for drug delivery applications. Gb3 is the ligand of several lectins, including the bacterial lectin Shiga toxin B-subunit (StxB) from *Shigella dysenteriae* ([27]. The Gb3 binding of StxB was used to probe the formation of Gb3 at the membrane surface of GUVs and SLBs. Moreover, complementary molecular dynamics simulations on glycosphingolipid-containing membranes were used to rationalize the experimental data [28].

## 2. Materials and Methods

### 2.1. Materials

C8 lactosyl(β)-ceramide (LacCer), 1,2-dioleoyl-sn-glycero-3-phosphocholine (DOPC) and cholesterol were obtained from Avanti Polar Lipids (Alabaster, AL, USA). Atto 647N 1,2-dioleoyl-sn-glycero-3-phosphoethanolamine (DOPE) was purchased from Sigma-Aldrich (Darmstadt, Germany). Globotriaosylceramide (Gb3) was obtained from Matreya (State College, PA, USA). Shiga toxin B-subunit (StxB) was kindly provided by enGenes Biotech (Vienna, Austria). UDP-Glo Glycosyltransferase assay kit and ultra-pure UDP-Galactose (UDP-Gal) were from Promega (Charbonnières-les-Bain, France). UDP-Gal for QCM-D analysis was purchased from Carbosynth (Compton, UK). HisTrap FF column was purchased from Cytiva (Marlborough, MA, USA) and Superdex 200 Increase 10/300 GL from GE Healthcare (Chicago, MA, USA). All other chemical reagents were of analytical or liquid chromatography grade. 

### 2.2. LgtC Production

The expression and purification of LgtC are based on previously published protocols [29,30]. The LgtC gene from *Neisseria meningitidis* was designed with 25 amino acids truncated at the C-terminus [30]. Codons were optimized for *E. coli* expression, and the mutations C128S, C174S and T273A were introduced in the LgtC sequence. These mutations were reported to improve expression levels of the protein without compromising the enzyme activity [29]. The gene was inserted into the pET-TEV vector using the restriction sites Ndel and Xhol, forming the LgtC-TEV construct. The gene construct contains a C-terminal (His)_6_-tag. LgtC-TEV vector was transformed into the expression strain *E. coli* BL21 (DE3). Cell culture was grown in LB media to an optical density (OD_600nm_) of 0.7–0.8 before induction with 0.5 mM isopropyl β-D-1-thiogalactopyranoside (IPTG). Protein expression occurred overnight at 16 °C, shaking at 180 rpm, before cells were harvested. The cell pellet was resuspended in buffer A (20 mM Tris, 1 mM DTT, 500 mM NaCl, 10 mM imidazole, pH 7.5), and cells were lysed using Cell Disruptor CSL (Constant Systems, Ltd., Daventry, UK) at 1.9 kbar. The cell lysate was then centrifuged at 24,000× *g*, 4 °C, for 30 min. The supernatant was collected and clarified through a 0.22 μm filter and purified by affinity chromatography using a HisTrap FF column with buffer A, and protein elution was performed with a 10–500 mM imidazole gradient. Fractions containing LgtC were collected and further purified by size exclusion chromatography on a Superdex 200 Increase 10/300 GL, in 20 mM Tris, 5 mM TCEP, pH 8.5. This buffer was optimized for protein stability and long-term protein storage at −20 °C. TCEP instead of DTT was preferred due to its higher stability in solution. 

### 2.3. Enzyme Activity Using UDP-Glo Glycosyltransferase Assay

The UDP-Glo assay quantifies the amount of UDP product formed from a glycosyltransferase reaction. The principle of the assay is to convert UDP to ATP to generate light in a luciferase reaction. A white 96-well microplate (Ref. 655074, Greiner Bio-One) containing the nucleotide detection reagent was used to stop the glycosyltransferase reaction and initiate luminescence. Luminescence was read using the Spark microplate reader (TECAN). The enzymatic rate was determined using a UDP standard curve, and activity was expressed, depending on the data analysis, as relative light units (RLU), as specific enzyme activity (nmol/min/mg protein) or as a reaction rate (μM/min). Assays were carried out in triplicates. Control reactions were performed in the absence of an acceptor.

Enzyme activity with LacCer acceptor was performed as follows. We used a LacCer variant with a short C8 acyl chain, which is more soluble than naturally occurring LacCer [29]. The required amount of C8 LacCer in chloroform:methanol (2:1) was added to a glass tube and dried under nitrogen for 20 min. The lipid was resolubilized in 30 µL of reaction buffer (see below). The LacCer solution was sonicated at 35 kHz in an iced water bath sonicator (Bioblock Scientific) to form micelles for 10 min. LgtC was added to the reaction, and the addition of UDP-Gal initiated the enzyme reaction. The enzymatic reaction (30 µL final volume) contained 20 mM HEPES, 1 mM MnCl_2_, 1 mM DTT, 250 μM UDP-Gal, varying lactosylceramide concentrations and 10 ng/μL (0.29 μM) LgtC at pH 7.5. The enzyme reaction proceeded for 10 min or longer where appropriate. 25 μL of the reaction was mixed with 25 μL nucleotide detection reagent. The plate was covered with foil, shaken for 30 s and incubated for 1 h at room temperature before measuring the luminescence. Enzyme activity with the acceptor lactose was performed in the same buffer conditions in a final volume of 100 µL. At the end of the reaction, 25 μL was transferred into a 96-well plate and mixed with 25 μL nucleotide detection reagent, as described above. All enzyme reactions were performed in triplicates at room temperature.

### 2.4. Thin Layer Chromatography (TLC) 

TLC was used to detect the formation of Gb3 by chemical staining following the procedure previously described [29]. An amount of 10 µL of enzyme reaction was spotted on a TLC Silica gel 60 F254 (Supelco) and ran using a chloroform:methanol: water (65:35:5) eluent. A sulphuric acid-orcinol reagent was used to stain the LacCer and Gb3 products. As a commercial C8-Gb3 is unavailable, only C8-LacCer was used as a negative control. C8-LacCer migrated with a Rf value of 0.61 and C8-Gb3 with a Rf value of 0.49. 

### 2.5. Giant Unilamellar Vesicles (GUVs) 

The GUVs were formed by a classical electro-formation protocol, as previously described [31]. In brief, solutions of lipids at a concentration of 0.5 mg/mL were composed of DOPC, cholesterol, Atto 647N DOPE and the glycosphingolipid C8-LacCer, at a molar ratio (mol-%) of 29.7:30:0.3:40 or Gb3 at 64.7:30:0.3:5. Solutions were prepared in chloroform and spread on indium tin oxide (ITO) covered glass slides. For removing the residual solvent, the slides were incubated under vacuum for at least 1 h. A chamber was assembled with two slides, filled with 318 mOsm·L^−1^ sucrose solution as formation buffer and an AC electrical field with a voltage of 1 V·mm^−1^ was applied (to the chamber) for 2.5 h at room temperature. The LgtC reaction on 40 mol-% LacCer GUVs was initiated in 20 mM HEPES, pH 7.4, 1 mM MnCl_2_, 1 mM DTT, 100 ng/μL LgtC (2.9 µM), 0.5 mM UDP-Gal and incubated for 1 h. GUVs were observed in hand-built chambers using 318 mOsm·L^−1^ PBS as an imaging buffer. Images were acquired by confocal fluorescence microscopy (Nikon Eclipse Ti-E inverted microscope using a Nikon A1R confocal laser scanning system with laser lines: 405 nm, 488 nm, 561 nm and 640 nm; 60 oil immersion objective, NA = 1.49; Nikon Instruments, Inc., Melville, NY, USA) and analyzed using NIS Elements software (NIS Elements Confocal 5.20, Nikon Instruments, Inc.) and ImageJ (Fiji win64, Open source).

### 2.6. Quartz Crystal Microbalance with Dissipation Monitoring (QCM-D)

QCM-D measurements on silica-coated sensors (QSX303; Biolin Scientific, Västra Frölunda, Sweden) were performed with a Q-Sense E4 system (Biolin Scientific) equipped with 4 independent flow modules, connected to a syringe pump (Legato; World Precision Instruments, Stevenage, UK) to deliver a fluid flow of 20 µL/min. The working temperature was set to 24 °C. Changes in resonance frequency (Δ*fi*) and dissipation (Δ*Di*) were acquired from six overtones (*i* = 3, 5, 7, 9, 11 and 13, corresponding to resonance frequencies of *fi* ≈ 5, 15, 25, 35, 45, 55 and 65 MHz). Results from the overtones *i* = 3 are presented unless stated otherwise, and frequency shifts are presented normalized by the overtone number (Δ*F* = Δ*fi*/*i*). All other overtones provided qualitatively similar data. 

### 2.7. Small Unilamellar Vesicles (SUVs) 

Small unilamellar vesicles (SUVs) containing DOPC and C8-LacCer were prepared as previously described [19], with modifications. Briefly, lipids in chloroform were mixed at desired molar ratios (100:0, 95:5 and 80:20) at a total amount of 5 µmol and dried under a stream of nitrogen gas followed by drying in a vacuum desiccator for 2 h. The lipid mixtures were resuspended in a working buffer at 2 mg/mL and homogenized by five cycles of freezing, thawing and vortexing. The lipid suspensions were subjected to tip sonication in pulse mode (1 s on/1 s off) for 15 min with refrigeration to obtain SUVs. The SUV suspension was centrifuged at 12,100× *g* for 10 min to remove titanium debris (shed from the sonicator tip) and stored at 4 °C under nitrogen gas until use. QCM-D sensors were pre-treated in UV/ozone (30 min), placed in the flow chamber and installed in the flow modules of the Q-Sense E4 apparatus. Following the acquisition of a baseline in HBS buffer (10 mM HEPES, 150 mM NaCl, pH 7.4), SLBs were formed on the sensor surface by the flow of 50 μg/mL liposomes until frequency and dissipation were stable. SLBs were equilibrated first in HBS buffer and then in reaction buffer (RB; 20 mM HEPES, 0.5 mM MnCl_2_, 4.4 mM Tris, 1.1 mM TCEP, pH 7.4), which was matched in content with the LgtC storage buffer to avoid solution effects on the QCM-D response upon starting the enzyme reaction. The RB buffer contained MnCl_2_, which is required for LgtC activity [30], and DTT was added to enhance enzyme stability. Enzyme reactions were initiated in RB containing 250 µM UDP-Gal and desired concentrations of LgtC (1.72 μM or 17.2 μM). SLBs were washed with RB to stop the enzyme reaction and then with HBS for further analysis. A 6.5 µM flow of StxB in HBS was applied, and StxB binding was observed to report for Gb3 in the SLBs.

### 2.8. Computer Simulations 

The initial structure for simulations was created based on the X-ray structure of LgtC with donor and acceptor analogs (PDB code: 1GA8) [30]. The protein with 25 amino acids truncated at the C-terminus contained the mutations C128S and C174S. In addition, Met appeared instead of Mse over the protein sequence by replacing the Se atoms with S atoms. The missing residues in the PDB structure at positions 218–221 were added and refined using the Modeller9.15 programme [32]. These manipulations aimed at bringing the in silico structure to the experimental one. Furthermore, for calculations, UDP 2-deoxy-2-fluoro-galactose and 4′-deoxylactose lactose in the X-ray structure were converted into UDP-Gal and lactose, respectively. 

The lipid bilayer consisting of DOPC was built up using CHARMM-GUI [33,34]. LacCer (18:1/16:0) molecules were added to the “upper” leaflet of the membrane to have 10 mol-% of LacCer over the total amount of lipids in both layers (or 20% of the lipid content in the upper leaflet). The protein was attached to the membrane’s upper surface to keep the bound lactosyl moiety of LacCer as in the X-ray structure (1GA8) and was oriented in parallel to the membrane plane to avoid major steric conflicts with the membrane. 

### 2.9. Molecular Dynamics Simulations 

Two molecular systems were equilibrated during MD simulations: (1) protein with bound lactose and (2) protein pre-bound to LacCer anchored to the lipid membrane. Each system was placed in a cube with periodic boundary conditions and solvated with explicit water of TIP3P type. The charge in the resultant molecular systems was set as zero adding Na^+^ and Cl^-^ atoms in a proportion and amount to keep the ionic strength to 150 mM. Then the systems were energy minimized and equilibrated at 300 K and 1 bar pressure in an isothermal-isobaric ensemble before the production run. In the production run, 150 ns trajectories were accumulated for each system, ensuring that the root-mean-square deviations of the protein Cα-atoms reached a plateau over the trajectory. All the simulations were performed using CHARMM36 force field and GROMACS software [35,36]. The interaction energies between protein and lactose and protein and membrane were calculated based on the molecular mechanics with generalized Born and surface area solvation (MMGBSA) approximation, in which the free energy of binding is given as a combination of gas phase energy (MM), and the solvation effects, both electrostatic (GB) and non-electrostatic (SA). The entropy was calculated in the paradigm of interaction entropy [37]. The energy values were averaged over the last 50 ns of the trajectories. The calculations were carried out using the gmx_MMGBSA tool [38].

### 2.10. Molecular Docking 

The molecular docking was carried out using AutoDock4.2 [39]. The Lamarckian algorithm was used for the sampling of complexes based on X-ray structure. In the docking procedure, the receptor was kept rigid. In the ligand, all the O-H bands were allowed to rotate. The glycoside bonds were kept rigid, except for the case of the carbohydrate part of Gb3, where the terminal αGal(1-4)βGal were allowed to rotate; the rest was kept as for lactose in the bound state. The free energy of binding was calculated as

ΔE_binding_ = ΔE_vdW_ + ΔE_Hbond_ +ΔE_elec_ +ΔE_desolv_ + ΔE_tors_

where the individual terms denote contributions from: Δ E_vdW_—van der Waals contacts, ΔE_elec_—electrostatic interactions, ΔE_Hbond_—hydrogen bonds, ΔE_desolv_—desolvation and ΔE_tors_—entropic contribution. 

## 3. Results

### 3.1. LgtC Synthesizes Gb3 on LacCer-containing Micelles

A recombinant form of LgtC with a 25 amino acid C-terminal truncation was expressed in *E. coli* and purified as described in the experimental section. The C-terminal tail of LgtC is predicted to be an amphipathic α-helix to interact with the cytoplasmic membrane in bacteria (Appendix A). Its truncation increased protein solubility and yield while maintaining enzyme activity [30]. Activity tests were performed using the UDP-Glo glycosyltransferase assay, which detects the UDP product of the catalytic reaction. Enzymatic activity was first confirmed using lactose as the minimal acceptor. LacCer with a short C8 acyl chain was selected for enzyme assays in micelles. Optimal concentrations of UDP-Gal and LacCer were determined for kinetic analyses (Appendix B). Due to the precipitation of LacCer at concentrations above 200 µM, specific activities of LgtC were compared at 100 µM lactose and LacCer. The UDP-Gal concentration was saturating in all cases.

**Figure 2:**
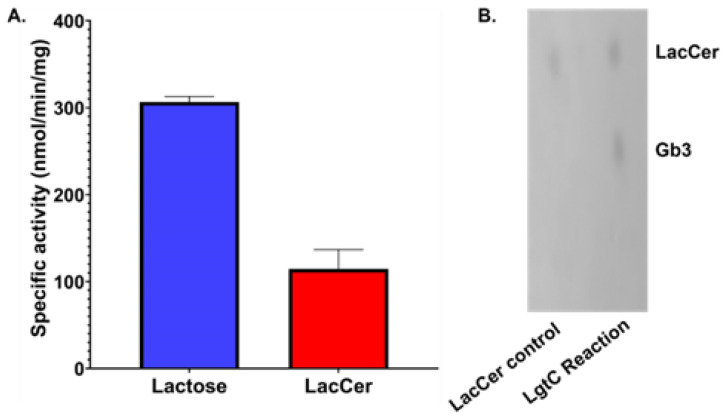
(**A**) LgtC activity on lactose and LacCer micelles. Conditions: 20 mM HEPES, pH 7.4, 1 mM MnCl_2_, 1 mM DTT, 100 µM lactose or LacCer, 250 µM UDP-Gal, 10 ng/μL (0.29 µM) LgtC. (**B**) Thin layer chromatography analysis of Gb3 formation. The enzyme reaction was run for 12 h to maximize the amount of Gb3 formed.

As represented in Figure 2A, LgtC showed 2.6-fold higher specific activity for the soluble acceptor lactose (307 ± 5 nmol/min/mg protein) compared to LacCer (117 ± 19 nmol/min/mg protein). Notably, the specific activity obtained with LacCer in micelles far exceeds that previously reported for LacCer solubilized using methyl-β-cyclodextrin (7.6 nmol/min/mg) [40], demonstrating the importance of the lipid organization for enzyme activity. From kinetic analyses of LgtC with varying LacCer concentrations (Appendix B), the catalytic efficiency (*kcat*/*K_M_*) of LgtC for LacCer in micelles was estimated to be 735 M^−1^·s^−1^, which is approximately 5-fold lower than the value reported for lactose (4000 M^−1^·s^−1^) [41].

Thin layer chromatography analysis was performed to confirm Gb3 formation (Figure 2B). The micelle enzyme reaction mixture showed the presence of spots, for the LacCer substrate and the Gb3 product, even after 12 h of enzyme reaction, indicating only partial conversion (estimated at ~50%) of LacCer to Gb3. The Rf value attributed to Gb3 is in perfect match with the spot that emerged for the Gb3 produced from C8-LacCer in another study [40]. One can reasonably postulate that, when present at a sufficiently high concentration, the Gb3 formed product blocks the access of LgtC to surrounding LacCer molecules in the micelles. Thus, although less efficient than with lactose, we demonstrated the ability of soluble LgtC to glycosylate LacCer, without any additives and in a supramolecular lipid assembly.

### 3.2. LgtC Activity on LacCer-Containing GUVs

**Figure 3:**
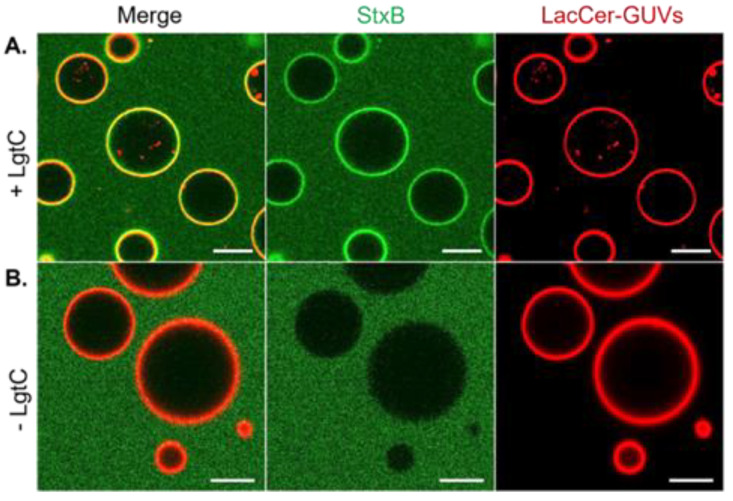
LgtC activity on LacCer-containing GUVs. Confocal images with GUVs visualized via the membrane marker Atto647N-DOPE (red), and Gb3 via StxB-AF488 (green) binding. (**A**) GUVs incubated with LgtC and UDP-Gal. Reaction conditions: 20 mM HEPES, pH 7.4, 1 mM MnCl_2_, 1 mM DTT, 100 ng/μL (2.9 µM) LgtC, 500 µM UDP-Gal and 40 mol-% LacCer-GUVs, incubation time 1 h at RT. Gb3 formation visualized by 500 nM StxB-AF488. (**B**) Negative control, without LgtC. Scale bars: 10 μm.

GUVs were formed from a lipid solution containing LacCer (40 mol-%), phosphatidylcholine (DOPC, 29.7 mol-%), cholesterol (30 mol-%) and the fluorescent membrane marker Atto647N-DOPE (0.3 mol-%), using the electro formation procedure [31]. The formed GUVs were stable and ranged from 10 µm to 50 µm. The enzyme reaction consisted of a 1 h incubation of LacCer-containing GUVs with LgtC and UDP-Gal at room temperature. Subsequently, GUVs were treated with the fluorescently labelled Shiga toxin B-subunit (StxB-AF488) and visualized by confocal microscopy. StxB binding was observed on GUVs incubated with LgtC and UDP-Gal, but not on control GUVs without LgtC, demonstrating the successful on-membrane conversion of LacCer into Gb3 (Figure 3).

### 3.3. Enzymatic LacCer Glycosylation Monitored on SLBs

**Figure 4:**
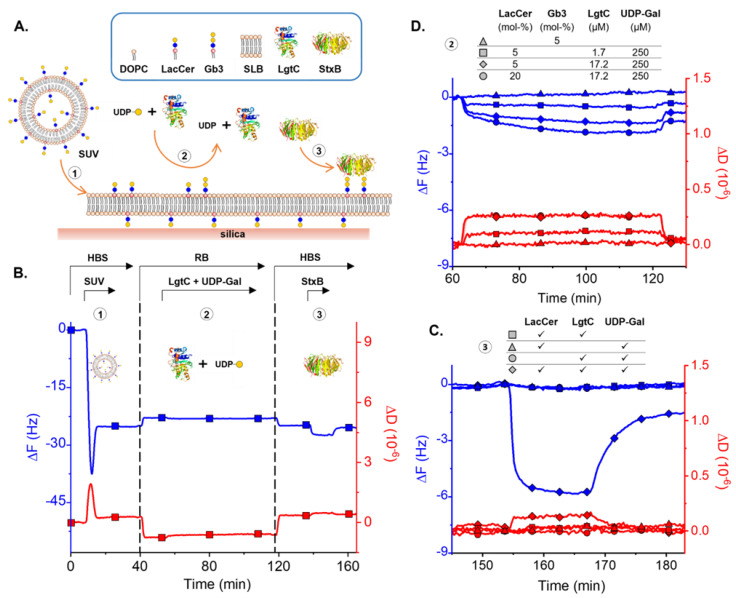
LgtC converts LacCer to Gb3 on supported lipid bilayers. (**A**) Schematic of the main steps of the assay: (1) supported lipid bilayer formation, (2) enzymatic conversion of LacCer into Gb3, (3) StxB binding reports on Gb3 content in the SLB. (**B**) Representative QCM-D data of the assay. A decrease in the frequency shift (Δ*F*, blue line) indicates mass binding to the surface (including hydrodynamically coupled solvent). The dissipation shift (Δ*D*, red line) provides a measure for the softness of the surface-confined film. Conditions: HBS, HEPES buffered saline (10 mM HEPES, 150 mM NaCl, pH 7.4); RB, reference buffer (20 mM HEPES, 0.5 mM MnCl_2_, 4.4 mM Tris, 1.1 mM TCEP, pH 7.4); SUV (50 μg/mL; 5 mol-% LacCer); LgtC (60 ng/µL, 1.7 µM); UDP-Gal (250 µM); StxB (6.5 μM); flow rate (20 µL/min). The arrows above the graph indicate the start and the duration of sample incubation and buffer exchange steps. (**C**) Zoom onto the StxB binding step of the experiment, the same as shown in B but at higher LgtC (600 ng/µL, 17.2 µM), including additional control experiments with either LacCer, LgtC or UDP-Gal missing, but otherwise identical (symbols as indicated above the graph). StxB was incubated from 155 to 167 min. (**D**) Direct monitoring of the enzyme reaction by QCM-D. LgtC on LacCer-containing SLBs at different reaction conditions. Conditions tested (symbols as indicated above the graph): 5 and 20 mol-% LacCer, 1.72 μM (60 ng/µL) and 17.2 μM (600 ng/µL) LgtC, reaction time 1 h. Control: 5 mol-% Gb3, run with buffer for 1 h.

To better understand the behavior of LgtC in a lipid bilayer membrane system, we investigated LacCer-containing SLB by quartz crystal microbalance (QCM-D). The concept of the assay is illustrated in Figure 4A. Small unilamellar vesicles (SUVs) were prepared from a lipid solution composed of 5 mol-% LacCer and 95 mol-% of DOPC, and SLBs were formed by the method of vesicle spreading on silica surfaces [19]. This method allows for the robust control of membrane composition. QCM-D was used to monitor successful SLB formation and the subsequent enzyme reaction and reporting steps (Figure 4B). The enzyme reaction was initiated by exposing the SLBs to a solution containing LgtC and UDP-Gal, under a constant flow rate. Next, SLBs were washed with buffer and StxB binding was used as a reporter for Gb3 produced. While the enzymatic reaction produced only very small QCM-D responses (vide infra), subsequent StxB binding reported the presence of Gb3 after co-incubating LacCer-containing SLBs with LgtC and UDP-Gal, indicating successful LacCer-to-Gb3 conversion on SLBs. At equilibrium, StxB bound to the enzyme-catalyzed LacCer surface with a frequency shift Δ*F* = 5.8 Hz, and a dissipation shift Δ*D* = 0.05 × 10^−6^, while there was no binding on surfaces in the absence of either LacCer, UDP-Gal or LgtC (Figure 4C). After rinsing with buffer, most bound StxB was removed from the surface. The binding of StxB likely reflects multivalent recognition of several Gb3 molecules by the StxB pentamer, harboring a total of 15 weak Gb3 binding sites [42,43]. Although avidity effects are apparent, they are not strong enough for stable binding (Appendix C).

The enzyme reaction was performed under different conditions to shed more light on the lipid membrane’s reaction mechanism. As shown in Figure 4D, a slight frequency shift at the start of the enzyme reaction is detected, caused by the interaction of LgtC with the membrane. Frequency shifts remained minor even when the LacCer content in the SLB was increased from 5 to 20 mol-% or when the LgtC concentration was varied from 1.72 µM to 17.2 µM. This indicates that very little enzyme is bound to the surface at any time and a short-lived interaction (transient binding) between LgtC and LacCer is sufficient for a productive enzyme reaction. Minor but significant decreases in the frequency (on the order of 1 Hz) were detectable during the enzyme reaction, demonstrating that QCM-D is sensitive to the enzymatic addition of a monosaccharide to LacCer. Indeed, the magnitude of the shifts (between −0.5 Hz and −2 Hz, depending on the conditions; Figure 4D) are comparable to Δ*F* ≈ −3 Hz predicted for a film thickness increase by 0.5 nm (i.e., equivalent to the size of a monosaccharide). 

From the kinetics of the frequency response for 17.2 µM LgtC and 5 mol-% LacCer (Figure 4D), one can estimate that the reaction is completed within less than 1 h. Further analysis of StxB binding as a function of the reaction time corroborates this estimate and indicates half-maximal binding within approximately 20 min (Appendix C). From these numbers, a catalytic efficiency of ~24 M^−1^·s^−1^ was estimated for LacCer in a lipid bilayer membrane context (see Appendix C for calculations). This is substantially less efficient than the published data for lactose (4000 M^−1^·s^−1^) [30]. However, along with estimates of the catalytic efficiency on micelles (735 M^−1^·s^−1^) (lying between SLB and lactose), this would suggest reduced access to the substrate in the context of membranes.

### 3.4. Computational Modeling Reveals Steric Hindrance between LgtC and the Lipid Bilayer

Molecular modeling simulations offer a valuable tool to gain both atomistic and thermodynamic parameters of protein–carbohydrate interactions in realistic environments [44]. Here, the geometry and thermodynamic parameters of LgtC binding to its ligands (UDP-Gal, lactose, LacCer, Gb3), in solution and on the membrane surface were calculated using molecular docking and molecular dynamics (MD) simulations (Appendix D). 

The lactose binding by LgtC in solution is weak with a *K_d_* in the mM range (15 mM). The enthalpic term of the binding energy resulting from the interaction between the lactosyl moiety of LacCer and LgtC on the membrane surface is weaker than in solution (the *K_d_* cannot be adequately estimated because the entropy is uncertain), despite additional contacts formed between LgtC and lipids. These data support the transient binding of LgtC observed by QCM-D.

**Figure 5:**
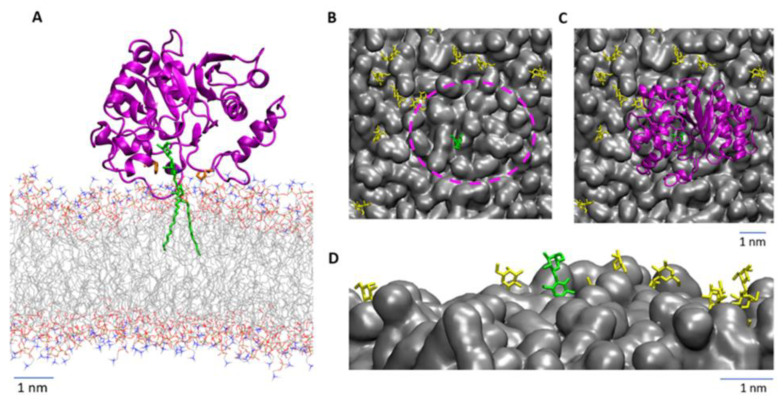
(**A**) Interaction of LgtC with LacCer on the lipid membrane surface. A cartoon represents LgtC, the LacCer as green sticks and DOPC lipids with color-coded atoms (C-grey, O-red, N-blue, H not shown for clarity). (**B**,**C**) Top view showing the LacCer distribution in the membrane surface plane, without (**B**) and with (**C**) bound LgtC to illustrate the membrane area around LacCer (dashed pink line) required for protein binding. (**D**) Snapshot from MD trajectory showing the orientation of the lactosyl head groups of LacCer in the DOPC membrane (side view). The LacCer molecule bound by the protein over the whole trajectory is colored green, and the other LacCer molecules are colored yellow and DOPC grey.

The topology of the active site involves the entry of both the Gal and Glc residues into the site, and such binding brings the protein into close contact with the membrane surface (Figure 5A and Appendix D). However, additional contacts formed between LgtC and lipids are not beneficial because of unfavorable desolvation energy. Furthermore, the relation between the binding pocket’s depth and the glycosphingolipid head’s height above the membrane implies that DOPC molecules surround LacCer with smaller polar heads to avoid steric conflict between the protein and the membrane (Figure 5B,C). This suggests that LgtC prefers LacCer molecules that are isolated rather than present within a glycolipid cluster, consistent with our observation of incomplete conversion in micelles (Figure 2B). According to the results of coarse-grained simulations, LacCer and Gb3 remain dispersed in the bulk of phospholipids in the absence of LgtC. Interestingly, the binding of LgtC to LacCer becomes possible when LacCer adopts a particular conformation in the sugar/aglycone junction and rises above the membrane surface (C1 of glucosyl residue is ~0.3 nm higher than those in non-bounded LacCer molecules; Figure 5D). Another report, based on NMR and molecular modeling, supports our findings, highlighting the role of the presentation of the glycan in establishing the essential binding contacts. In liposomes, the Gal residue in LacCer was not permanently exposed to allow the interaction with galectins to take place efficiently [45]. Thus, LgtC binding to the membrane would be regulated by selecting the appropriate conformation of LacCer and the lipids surrounding LacCer.

## 4. Conclusions

We demonstrated that a soluble form of LgtC can recognize the lactosyl moiety of LacCer and add a third sugar. The reduced catalytic efficiency of the enzyme on lipid membranes compared to what was observed in solution with lactose and LacCer micelles is explained by the membrane exerting a steric hindrance for the enzyme to efficiently accommodate the lactosyl moiety of LacCer in the active site. Our data showed LacCer needs to rise above the membrane for effective binding. From these experimental data, one can anticipate that the LgtC reaction will be favored with a longer lipid-bound oligosaccharide chain (three or more sugar residues) and disadvantaged by LacCer with longer fatty acid chains. It correlates with the natural activity of LgtC, which acts on LOS that have longer chain oligosaccharides.

Overall, a better understanding of the behavior of GTs in different membrane environments will allow better catalysts to be used as synthetic glycobiology tools to construct sophisticated glycocalyces. Concerning LgtC, future work should aim to increase its catalytic efficiency as a Gb3 producer. The role of the C-terminal amphiphilic tail of full-length LgtC can also be questioned. Would this increase the frequency of productive interactions with LacCer by retaining the enzyme on the membrane surface longer or forcing the enzyme deeper into the membrane? This remains to be determined. This work opens up a new avenue for the dynamic modulation of the glycolipid content in model membranes. This could be of interest, for example, to study the impact of glycolipids on dynamic membrane re-organization (e.g., clustering; modulation of lipid phase separation), and the dynamic modulation of protein binding or membrane–membrane interactions.

## Figures and Tables

**Figure A1:**
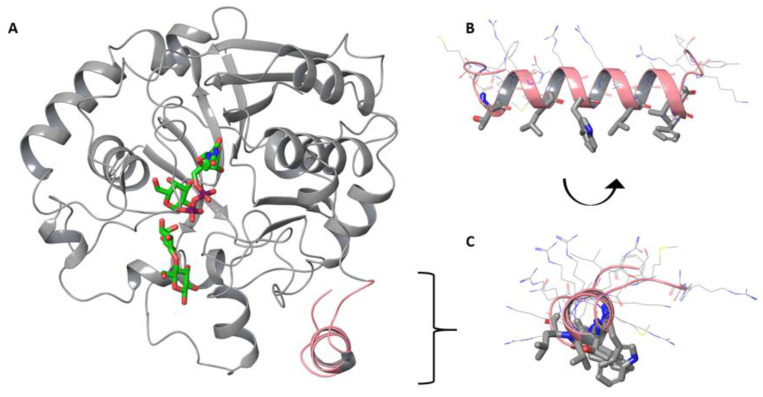
(**A**) The model of full-length LgtC predicted by AlphaFold2 with UDP-Gal and lactose obtained from 1GA8. (**B**,**C**) The C-terminal α-helix given in pink color with hydrophobic residues shown in thick lines. Color coding: grey for C atoms of the protein, green for C atoms of the ligands, blue for N atoms, red for O atoms.

**Figure A2:**
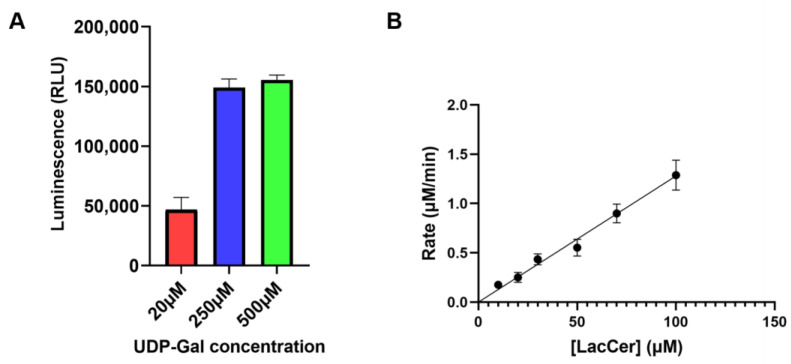
(**A**) LgtC activity at different UDP-Gal concentrations. Conditions: 20 mM HEPES, pH 7.4, 1 mM MnCl_2_, 1 mM DTT, 10 ng/μL (0.29 μM) LgtC, 0.1 mM LacCer, UDP-Gal (20, 250 and 500 μM). (**B**) LgtC activity at different LacCer concentrations. The solid line represents the best fit to the simplified Michaelis–Menten equation, yielding *k*_cat_[*E*]/*K*_M_ = 0.0128 min^−1^. Conditions: 10 ng/μL LgtC, 0.25 mM UDP-Gal.

**Figure A3:**
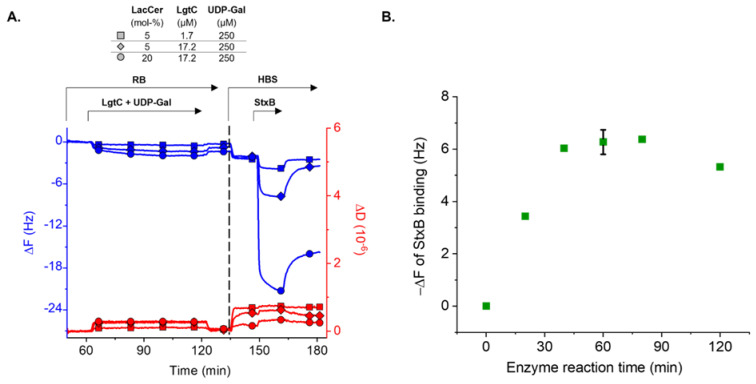
Parameters determining the reaction rate. (**A**) Representative QCM-D data of the enzyme reaction under different conditions (lines with symbols as indicated), and subsequent reporting of Gb3 by StxB binding. The net frequency changes (Δ*F*) after the enzyme reaction step were minor, ranging from −0.3 Hz (5 mol-% LacCer, 1.72 μM LgtC) to −0.8 Hz (5 mol-% LacCer, 17.2 μM LgtC) to −1.0 Hz (20 mol-% LacCer, 17.2 μM LgtC), and the net dissipation shifts (Δ*D*) were also small. The frequency shifts at equilibrium for StxB binding were −1.5 Hz, −5.8 Hz and −18.8 Hz, respectively. (**B**) Plot of −Δ*F* for equilibrium StxB binding (at 6.5 μM StxB; a measure of the Gb3 content in the SLB) vs. enzyme reaction time. Conditions: 5 mol-% LacCer, 17.2 μM LgtC and 0.25 mM UDP-Gal. The reaction is complete within approximately 60 min, leading to −Δ*F* = 6.5 ± 0.5 Hz (*n* = 3). Half maximum −Δ*F* is approximated to be at 20 min.

## Data Availability

The data supporting this study are available from the corresponding authors upon reasonable request.

## Appendix A

**LgtC protein sequence analysis**

The sequence used in this work is shown in black, the truncated C-terminal tail is in red:

MDIVFAADDNYAAYLCVAAKSVEAAHPDTEIRFHVLDAGISEANRAAVAANLRGGGGNIR-60

FIDVNPEDFAGFPLNIRHISITTYARLKLGEYIADCDKVLYLDIDVLVRDSLTPLWDTDL-120

GDNWLGASIDLFVERQEGYKQKIGMADGEYYFNAGVLLINLKKWRRHDIFKMSSEWVEQY-180

KDVMQYQDQDILNGLFKGGVCYANSRFNFMPTNYAFMANWFASRHTDPLYRDRTNTVMPV-240

AVSHYCGPAKPWHRDCTAWGAERFTELAGSLTTVPEEWRGKLAVPHRMFSTKRMLQRWRR-300

KLSARFLRKIY-311

The structure of the full-length protein was predicted using the AlphaFold2 server (available online: https://alphafold2.biodesign.ac.cn/, accessed on 1 May 2022) [46]. The terminal fragment within the residues 292–311 was predicted to form an α-helix with high confidence and the fragment 283–291 (underlined) is rather disordered with low confidence in the positioning (pLDDT 50–70). The α-helix is amphiphilic and has hydrophobic residues facing one side and a highly positively charged region facing the other side of the helix, thus predicting the monotopical location of the α-helix on the membrane (Figure A1).

## Appendix B

**LgtC activity on LacCer-containing micelles—optimizing assay conditions and estimating the catalytic efficiency**

As previously described, the *K*_M_ of LgtC for UDP-Gal is 18 μM [30]. However, this value was measured using lactose as the acceptor, which may change with LacCer as the acceptor. The activity of LgtC at different concentrations of UDP-Gal was measured to verify that future reactions with LacCer were at saturating concentrations of UDP-Gal. Figure A2A shows that there is almost no change in activity from 250 μM to 500 μM, indicating that reactions are at saturating conditions of UDP-Gal. Based on the activity obtained at 20 µM, one can estimate the *K*_M_ value for UDP-Gal slightly above this concentration. A concentration of a minimum of 250 µM was used in all subsequent enzyme assays.

A critical micelle concentration (CMC) of 150 nM for mixed LacCer (mixture of variable length acyl chain species) was previously estimated [47]. While the CMC of C8-LacCer would vary from the reported value for mixed LacCer, this information nevertheless allows for estimation of a lower limit for the formation of LacCer micelles. At ~200 μM LacCer, precipitation was observed. This is in line with the maximum solubility concentration observed with other short chain ceramides at 100 μM [48]. Although true saturating concentration of LacCer could not be achieved in the context of the Michaelis–Menten equation, estimation of catalytic efficiency was possible. As shown in Figure A2B, a linear response is obtained when varying the LacCer concentrations from 10 to 100 µM. This suggests that in this concentration range, we are at [LacCer] << *K*_M_ and therefore the Michaelis–Menten equation can be simplified to

$$v=v_{\max}\frac{\left[S\right]}{K_{M}+\left[S\right]}\approx \frac{V_{\max}\left[S\right]}{K_{M}}=\frac{k_{\mathrm{cat}}\left[E\right]\left[S\right]}{K_{M}}$$

with the reaction rate *v*, the maximal reaction rate *v*_max_, the substrate concentration [S] = [LacCer], the enzyme concentration [E] = [LgtC], and the turnover number *k*_cat_. From a fit to the data in Figure A2B, and considering [E] = 0.29 μM, we estimated the catalytic efficiency of LgtC on LacCer in micelles to *k*_cat_/*K*_M_ = 735 M^−1^·s^−1^.

## Appendix C

**LgtC activity on LacCer in SLB—Estimating the catalytic efficiency**

Since the enzyme reaction is performed at a saturating UDP-Gal concentration, the rate-limiting factors are the LacCer and LgtC concentrations, as demonstrated in Figure A3A. We monitored StxB binding by QCM-D as a function of the reaction time (with 17.2 µM LgtC and 5 mol-% LacCer) to estimate the catalytic efficiency of LgtC on LacCer in SLBs (Figure A3B).

The reaction was complete within approximately 60 min, leading to −Δ*F* = 6.5 ± 0.5 Hz (*n* = 3), and half-maximal frequency shift in StxB binding was reached after t_0.5 ≈ 20 min. From these data, a catalytic efficiency of 24 M^−1^·s^−1^ can be estimated using the simple model described below. Our model takes into account that enzyme binding and the enzymatic reaction taking place on surfaces, and that the affinity of LgtC for LacCer is low. We consider the enzymatic reaction:

E+S⇄ES→E+P.

The enzyme E is free to diffuse in the solution phase (and thus expressed in molar concentration), but the substrate S, the enzyme–substrate complex ES and the product P are confined to the surface (and thus expressed in molar surface density).

The reaction rate is determined as $v=k_{\mathrm{cat}}\left[\mathrm{ES}\right]$.

From the first order rate constant (also called turnover number), 𝑘_cat_ and the concentration of the enzyme–substrate complex [ES]. We can assume instantaneous chemical equilibrium at the surface, due to low affinity between LgtC and LacCer, and the surface density of ES is thus given by the Langmuir isotherm:

$$\left[\mathrm{ES}\right]={\left[S\right]}_{\mathrm{tot}}\frac{\left[E\right]}{K_{d}+\left[E\right]}$$

where *K*_d_ is the equilibrium dissociation constant of ES, and [S]_tot_ the total surface density of available substrate. In the limit of small enzyme concentrations ([E] ≪ *K*_d_) we can approximate *K*_d_ + [E] ≈ *K*_d_, leading to

$$v\approx \frac{k_{\mathrm{cat}}}{K_{d}}{\left[S\right]}_{\mathrm{tot}}\left[E\right]$$

In our assay (constant flow), the enzyme concentration in solution is maintained constant and equal to the enzyme concentration injected in the system ([E] = [E]_0_). Moreover, at the beginning of the reaction, the substrate concentration is close to the initial substrate concentration ([S]_tot_ ≈ [S]_0_). The initial reaction velocity thus is $v_{0}\approx \frac{k_{\mathrm{cat}}}{K_{d}}{\left[S\right]}_{0}{\left[E\right]}_{0}$, leading to

$$\frac{k_{\mathrm{cat}}}{K_{d}}\approx \frac{v_{0}}{{\left[S\right]}_{0}{\left[E\right]}_{0}}$$

With [E_0_] = 17.2 μM, and $v_{0}\approx \frac{0.5{\left[S\right]}_{0}}{t_{0.5}}\approx \frac{{\left[S\right]}_{0}}{40\;\min}$, we obtain a catalytic efficiency *k*_cat_/*K*_d_ ≈ 24 M^−1^·s^−1^. This value should be used as an order of magnitude estimate due to two rather crude approximations used to estimate *t*_0.5_: (i) the frequency shift is proportional to StxB surface density and (ii) the StxB surface density is proportional to Gb3 surface density [49].

## Appendix D. Lactose Binding in Solution

***LgtC—Molecular Modeling***

To estimate the binding energies of lactose to LgtC, the computational protocol was first verified against an experiment using α3GT (PDB code: 1GWV) that is specific to the same ligands, UDP-Gal and lactose, as LgtC [50]. The binding energy based on the docking score function between lactose and α3GT (Table A1) amounted to −4 kcal/mol, which was close to that measured by ITC −3.57 kcal/mol (the dissociation constant *K*_d_ amounted to 2.68 mM) [50]. For LgtC, the binding energy (based on the docking score function) between the protein and lactose was around −2.5 kcal/mol (Table A1), revealing quite weak affinity with a *K*_d_ in the mM range (15 mM), comparable to the binding affinity of lactose to α3GT. The affinity of LgtC to its Gb3 product, αGal(1–4)βGal(1–4)βGlc, estimated by molecular docking, was also weak, the binding energy was around −3 kcal/mol and the *K*_d_ remained in the mM range (6 mM). The results of the docking of lactose or Gb3 to LgtC, in the presence of UDP-Gal or UDP, showed the same calculated affinities. Therefore, in the MD simulations the binding of lactose to LgtC without UDP-Gal was considered.

During the MD trajectory, the LgtC–lactose complex in solution remained stable. The MMGBSA binding energy is overestimated compared to that estimated based on docking score function (Table A1). Although the entropy term matches perfectly with the docking results, the enthalpy term is overestimated, bringing *K*_d_ of the complex to the µM range.

***Lactose Binding in the Membrane***

To obtain insights into the binding mode and binding energies of LgtC to the membrane, the protein was pre-bound to LacCer anchored into the membrane and the system was equilibrated during the MD trajectory. The analysis revealed that the binding of LgtC to LacCer on the membrane surface brings the protein into close proximity to the bilayer so that the protein protrudes up to 4 nm above the membrane surface. The contacts between the protein and the lactosyl moiety of LacCer are similar to those observed in the complex with lactose [30]: residues of the active site I76, H78, I79, D130, V133, N153, Y186 and Q189 embed the galactosyl residue and residues F132, P211, T212 and P248 contribute to the binding the glucosyl residue. In addition to the contacts between LgtC and LacCer, numerous contacts between LgtC and DOPC molecules on the membrane surface are formed. In addition to the contacts between LgtC and LacCer, numerous contacts between LgtC and DOPC molecules on the membrane surface are formed involving the residues F132, R135, Q136, Y214, A215, F216, M217, A218, N219, W220, F221, R224, P248, D255 and T257. In contrast, the C-terminus (L282) of the truncated LgtC tends to elevate and does not interact with the membrane (potentially, the amphiphilic helix, absent in our construction, could retain the C-terminal domain of the protein closer to the membrane surface). The orientation of the protein is restricted by the membrane, which in turn implies some restrictions on the conformation of LacCer recognized by LgtC. In this conformation, the (phi, psi) angles of βGal(1-4)βGlc glycosidic bond counting as O5-C1-O4-C4 and C1-O4-C4-C5 are (−74 ± 10, −127 ± 9). For the linkage of βGlc to Cer, three angles, namely O5-C1-O1-C1S, C1-O1-C1S-C2S and O1-C1S-C2S-C3S, are restricted by the values (−70 ± 8, 121 ± 15, 180 ± 8). The latter is a rare conformation which is populated only 5% of time in non-bonded LacCer but is retained longer due to the protein binding.

The additional contacts of LgtC to the membrane surface may contribute to the binding energy. According to this assumption, in the binding energy of the protein to the membrane over the MD trajectory, the term of gas phase protein–membrane interaction increases, when compared to that for protein–disaccharide interactions in solution. However, in the MMGBSA approximation, this increase is compensated by rather unfavorable energy of desolvation, such that the resultant enthalpy of LgtC binding to the membrane became lower than that for protein-lactose in solution. The entropy term, calculated by the interaction entropy method, is not reliable because of high fluctuations in the interaction energy between the protein and the membrane over time, while subtle energy fluctuations are a prerequisite for the application of the method [37].

**Table A1:** Thermodynamic parameters of LgtC binding to its ligands calculated using molecular docking and molecular dynamics (all values are in kcal/mol).

	Docking			MD				
	ΔG	ΔH	−TΔS	ΔG	ΔH			−TΔS
					Total	Gas	Desolv	
LgtC/βGal(1−4)βGlc	−2.5	−6.0	+3.6	−8.9 ±6.7	−12.9 ±5.3	−49.5 ±4.5	36.6 ±2.5	+3.9 ±4.2
LgtC+UDP−Gal/βGal(1−4)βGlc	−2.6	−6.2	+3.6					
LgtC/αGal(1−4)βGal(1−4)βGlc	−3.0	−8.4	+5.4					
LgtC+UDP/ αGal(1−4)βGal(1−4)βGlc	−3.2	−8.5	+5.4					
LgtC/LacCer in membrane ^(a)^					−2.9 ±109	−528 ±89	525 ±64	NA
α3GT+UDP/βGal(1−4)βGlc ^(b)^	−4.0	−7.6	+3.6					

^(a)^ The whole membrane with LacCer bound to LgtC was considered as a ligand to estimate the binding energy. The SD value largely prevailing over the mean value of ΔH_total_ is due to large mean values annihilating upon summing ΔH_gas_ and ΔH_desolv_. ^(b)^ The related ITC data calculated for α3GT for the binding of lactose in the presence of UDP were *K*_d_ = 2.68 mM, ΔG = −3.57 kcal/mol, ΔH = −9.47 kcal/mol, −TΔS = +5.88 kcal/mol [50].
